# Chemistry, Properties, and Patterning of Transparent and Conductive Materials

**DOI:** 10.1021/acs.jpcc.5c05925

**Published:** 2025-10-18

**Authors:** Keidy L. Matos, Anthony J. Russo, Adam B. Braunschweig

**Affiliations:** † Advanced Science Research Center, Graduate Center, City University of New York, 85 St. Nicholas Terrace, New York, New York 10031, United States; ‡ Department of Chemistry, 217480 Hunter College, 695 Park Avenue, New York, New York 10065, United States; § PhD Program in Chemistry, Graduate Center, City University of New York, 365 fifth Avenue, New York, New York 10016, United States; ∥ PhD Program in Biochemistry, Graduate Center, City University of New York, 365 fifth Avenue, New York, New York 10016, United States; ⊥ Hunter College High School, 71 E 94th Street, New York, New York 10128, United States

## Abstract

Transparent and conductive (T/C) wires and patterns are essential components in modern optoelectronic devices, including touchscreens, solar panels, smart windows, and wearable sensors. These technologies rely on materials that simultaneously transmit visible light, conduct electricity, and can be patterned, requirements that pose substantial chemical, material, and processing challenges. Indium tin oxide, the longstanding industry standard for T/C applications, offers high transparency and low sheet resistance, but suffers from brittleness and restricted stretchability. These limitations have spurred intense research into alternative T/C materials, including metallic nanowires, carbon-based conductors, and conductive polymers, each offering unique advantages related to conductivity, flexibility, environmental stability, and fabrication compatibility. This perspective provides an overview of the properties demanded of T/C materials and recent advances in new T/C chemistry and fabrication techniques used to create T/C patterns.

## Graphical abstract

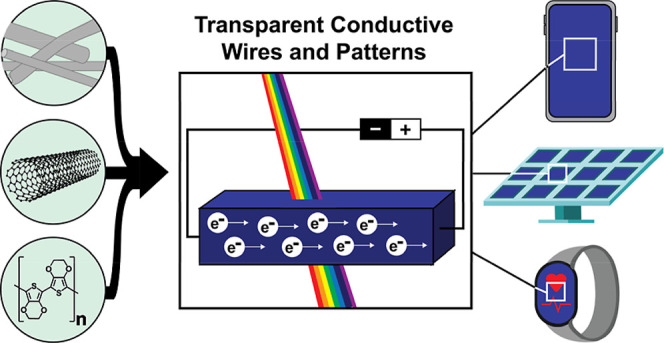

## Introduction

Wires and patterns that are both transparent and conductive (T/C) are an indispensable modern technology that are essential components in optoelectronic devices like displays, smart windows, wearable sensors, augmented/virtual reality (AR/VR) goggles, and solar cells^1,2^ (Figure 1). These patterns utilize materials that combine transparency in the visible spectrum with electrical conductivity. In displays, such as those used in smartphones, laptops, tablets, AR/VR goggles, and televisions, T/C patterns serve as electrodes that transmit electrical signals that cause pixels to illuminate (Figure 1A). These electrodes are typically patterned into fine grids and must maintain high optical transmittance, while offering sufficient conductivity to register user inputs and deliver rapid electrical signals. Alternatively, in solar cells, T/C materials act as the front electrode, allowing sunlight to pass through, while efficiently collecting and transporting photogenerated charges^3^ (Figure 1B). For smart windows,^4,5^ T/C films control the flow of current to electrochromic layers, allowing the glass to change its opacity or color. Similarly, wearable sensors^6,7^ (Figure 1C) rely on flexible T/C wires for monitoring physiological signals, such as heart rate, body temperature, and motion.^8^ The architecture of these sensors often includes transparent interface layers, stretchable current collectors, and stretchable anodes, semiconductors, and cathodes. These stacked components are designed to maintain conductivity under strain, often using mesh geometries or nanomaterials embedded in elastic matrices. The transparent interface layers act as optical windows and electrical contacts, while the stretchable components enable continuous monitoring of motion, temperature, or biopotentials during wear. For all of these applications, T/C patterns on rigid or flexible substrates must maintain high optical clarity and often touch sensitivity and the ability to individually address and manipulate each pixel’s brightness, color, and timing.

**Figure 1:**
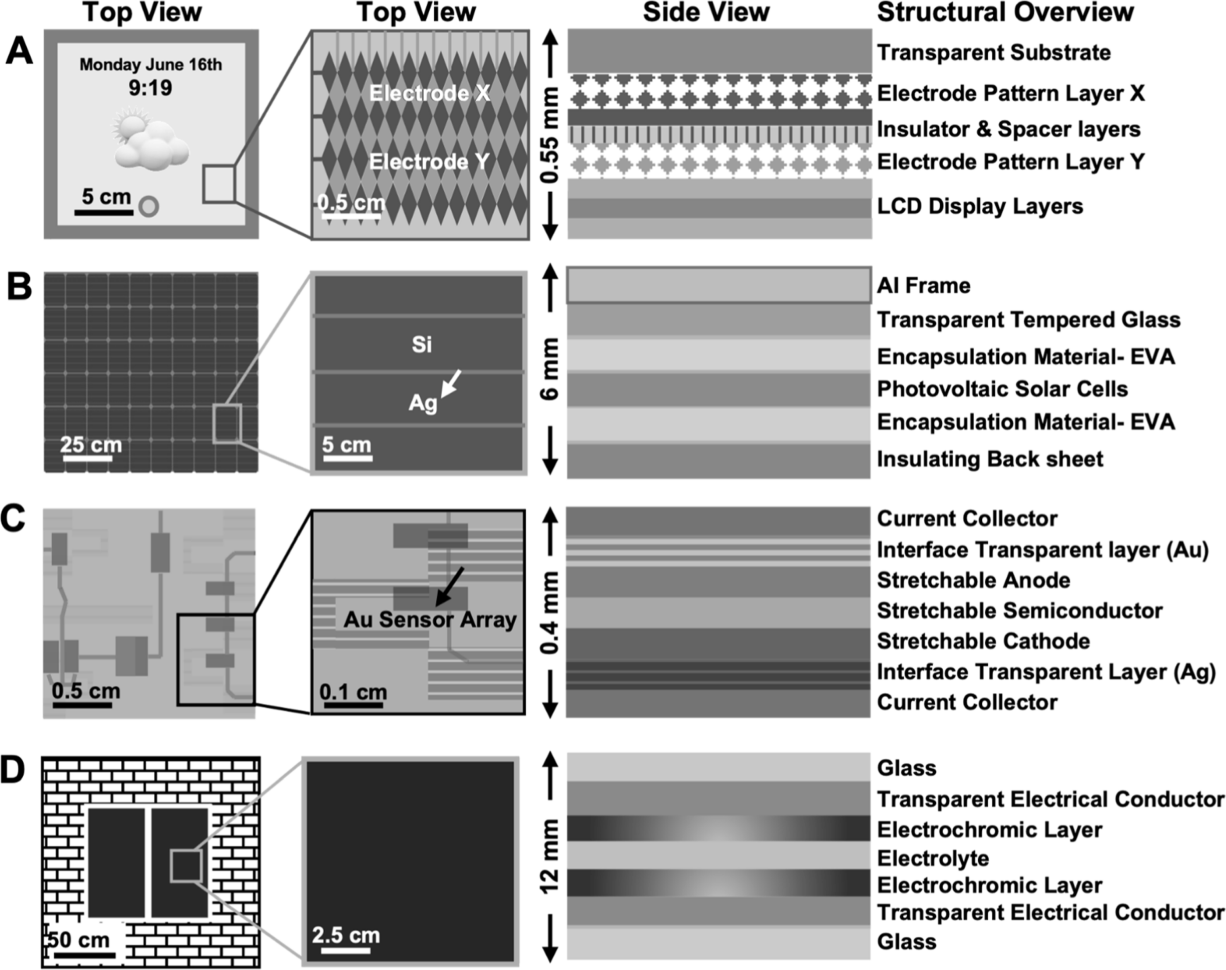
Layer-by-layer breakdown of optoelectronic devices containing transparent/conductive (T/C) materials. (A) Left: top view of a touchscreen device. Middle: top view of the transparent electrode X–Y patterned grid within touchscreens. Right: cross section of the layers that make up a touchscreen. (B) Left: top view of a common solar panel device. Middle: top view of the photovoltaic solar cell within solar panels. Right: cross section view of the layers that make up a solar panel. (C) Left: top view of a wearable electronic device, consisting of various circuits and sensors. Middle: top view of a sensor array within a wearable electronic sensor. Right: cross section of the layers that make up a wearable sensor. (D) Illustration of a smart window. Zoomed in view of a smart transparent window. Right: cross section of the layers that make up a smart window.

To meet the demands of next-generation technologies, T/C materials must satisfy several stringent performance criteria, which vary depending on the application (Table 1), motivating the development of new materials and fabrication strategies for T/C layers, where the properties can be precisely tailored to suit the demands of the technology.^9,10^ T/C materials are evaluated based on optical transmittance (*T*), sheet resistance (*R*_s_), work function (Φ), mechanical flexibility, chemical and environmental stability, and manufacturing scalability.^11^ For applications such as touchscreens and flexible displays, T/C materials should exhibit visible light transmittance above 80%, while maintaining an *R*_s_ < 500 Ω·sq^–1^.^9^ For more demanding applications, such as transparent antennas or high-performance sensors, target *R*_s_ decreases to <10 Ω·sq^–1^ without sacrificing transparency.^10^

**Table 1:** Performance Demands of T/C Layers for Optoelectronic Devices

Application	Transmittance 400–800 nm (%)	Sheet resistance (Ω·sq^–1^)	Work function (eV)	Flexibility requirements	Environmental stability	Manufacturing requirements
Touchscreens & displays	≥85	<500	4.7–5.2	bending radius <5 mm, >10,000 cycles	high (humidity, UV, heat resistance)	scalable, low-cost (R2R or solution processing)
Flexible sensors	≥85	<50	4.5–5.0	stretchable (>50% strain)	moderate (moisture/oxidation resistance)	solution processing on polymer substrates
Smart windows	≥75	<400	4.3–4.9	flexible for curved surfaces, minimal cycling	high (UV, heat resistance)	large-area processing
Solar cells	≥85	<30	4.2–5.3	minimal flexibility (for rigid panels)	very high (UV, heat, moisture)	cost-effective for large-area panels

In designing T/C materials, it is important to distinguish between roles that demand primarily capacitive behavior versus those requiring charge-transfer (faradaic or continuous conduction).^12^ Capacitive applications include T/C films for touch screens, transparent heaters, capacitive sensors, or antenna structures, and the materials selection criteria emphasize very high optical transparency, low sheet resistance, mechanical flexibility, and minimal chemical reactivity.^13,14^ In such contexts, the conductor’s ability to store and release charge via electric double layer capacitance or via surface redox reactions is sufficient, and stability under strain or environmental exposure can be as critical as raw conductivity.^15^ By contrast, charge-transfer applications (photovoltaics, LEDs, electrochromic devices, or electrodes in energy conversion/storage) require continuous injection and extraction of electrons (and possibly ions), steady energy level alignment, high carrier mobility, and often significant chemical and interfacial stability.^16,17^ In these applications, trade-offs in selecting the correct materials include balancing optical transmittance and conductivity and the film’s ability to sustain high current densities and manage interfacial contact losses and recombination.^18^

In addition to *T* and *R*_s_, Φ is a critical parameter involved in selecting a T/C material for a particular application, as Φ determines energy level alignment with adjacent layers and directly influences charge injection or extraction efficiency.^19^ Optimal Φ values vary by application: for example, in organic light-emitting diodes (OLEDs) and LEDs (Figure 2A), the T/C material’s Φ should align closely with the highest occupied molecular orbital (HOMO) of the hole transport layer (typically ∼4.8–5.2 eV)^20,21^ to facilitate efficient hole injection. Holes are injected from the T/C anode into the HOMO while electrons are taken from the metal cathode into the lowest unoccupied molecular orbital (LUMO). Charge recombination happens in the electron accepting emitting layer, which then emits a photon (*hv*).^22^ The efficiency of this process depends on the alignment of the transparent anode and metal cathode’s Φ, which determine the position of the Fermi level (*E*_F_) with respect to the HOMO and LUMO, with their energies being determined with reference to the vacuum level (*E*_vac_). In solar cells (Figure 2B), Φ values are chosen to align with the donor or acceptor energy levels (often ∼4.2–4.8 eV for electron-collecting cathodes and ∼5.0–5.3 eV for hole-collecting anodes)^23^ to minimize energy barriers and recombination losses. Here, incoming photons generate excitons that separate at the donor–acceptor interface, with holes moving toward the T/C anode and electrons being collected at the metal cathode.^24^ In both LEDs and solar cells, the Φ alignment of the electrodes relative to the positions of *E*_F_ and *E*_vac_ define the driving forces for charge extraction and separation, ensuring that carriers can move efficiently through the semiconductor layer and across the electrodes.

**Figure 2:**
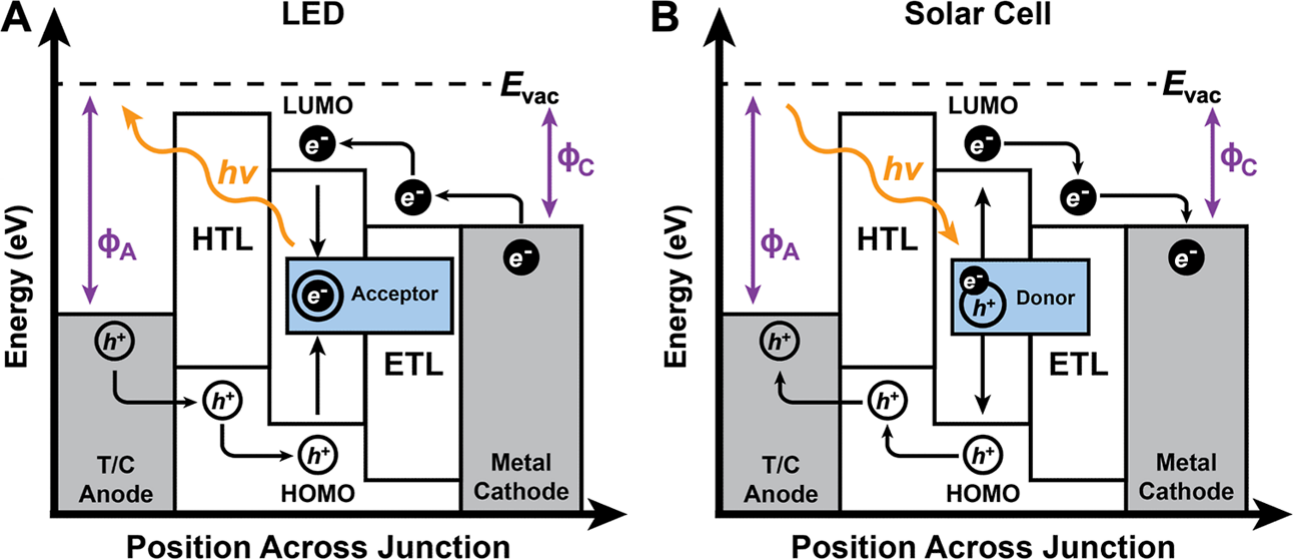
Energy level diagrams of (A) LEDs, for enhanced hole injection and light emission (*hv*). (B) Solar cells, efficient carrier separation and collection. *h* ^+^ represent the holes, *e* ^–^ the electrons, HTL is the hole transport layer, ETL is the electron transport layer, *E*_vac_ is the vacuum level, Φ_A_ is the work function of the T/C anode relative to the *E*_vac_ and Φ_C_ is the work function of the metal cathode relative to the *E*_vac_.

Mechanical flexibility and durability are also important properties to consider for devices such as wearable sensors,^6^ stretchable electronics,^7,25^ and flexible displays.^26–28^ For these applications, T/C materials must tolerate repeated bending, stretching, or folding for thousands of cycles without significant loss of conductivity. Long-term environmental stability is also critical,^29^ requiring resistance to oxidation,^30^ humidity,^31^ UV exposure,^32^ and thermal cycling.^33^ Finally, for widespread adoption, T/C materials should be compatible with scalable and cost-effective manufacturing processes, such as roll-to-roll (R2R) printing,^34^ solution processing,^35^ and low-temperature deposition,^36^ enabling integration onto diverse substrates, including glass, plastics, and textiles. Additionally, T/C devices, particularly those requiring high resolution or complex architectures, still require fabrication in cleanroom environments for their integration into devices, where controlled conditions ensure high reproducibility.

The market for T/C materials is substantial and continues to grow as technologies like AR/VR systems, solar panels, and interactive displays become more widely adopted.^28^ This growth is driven by sustained demand in consumer electronics, renewable energy, healthcare, and advanced optics. The AR/VR sectors, for example, are experiencing explosive growth across industries including gaming, defense, education, and medicine.^37^ Similarly, the solar industry has seen remarkable expansion, with installations and capacity nearly doubling in recent years, fueled by advances in photovoltaic technology and global climate initiatives.^38,39^ Touchscreen devices, now ubiquitous in smartphones, tablets, and public kiosks, have also undergone rapid evolution, moving toward more flexible, high resolution, and responsive formats.^40^ These market trends highlight not only the growing commercial relevance of T/C materials, but also the increasing performance and fabrication demands placed upon them. Reflecting this momentum, the number of publications appearing under the search term “transparent conductive wires and patterns” in the Science Direct search engine has increased from 61 in 2004 to a total of 5301 in 2024 (Figure 3A). From a commercial perspective, the global market for transparent conductive materials was valued at $5.07 billion (B) in 2023 as reported by the analysis from Market Research Future.^41^ This value has been projected to grow at a compound annual growth rate (CAGR) of 7.46%, reaching $12B by 2035 (Figure 3B). This growth is attributed to the increasing demand across electronics, renewable energy, and automotive markets, and by innovations in T/C technologies.

**Figure 3:**
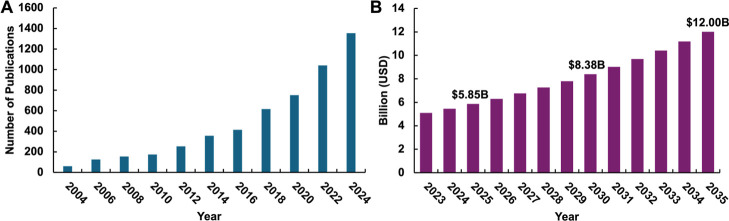
(A) “Transparent conductive wires and patterns” -related publication from 2004 to 2024 in the Science Direct search engines. (B) Projection of market size of transparent conductive materials from 2023 to 2035 calculated with a CAGR of 7.46%.^41^

Several reviews have previously examined the development of T/C materials, focusing on topics such as material selection,^2,26^ deposition techniques,^10,11,42^ and relationships between material composition and the resulting electrical/optical performance.^33^ However, since the last major review in 2018,^42^ the number of publications has significantly increased, demonstrating that the field has rapidly evolved with the emergence of novel materials, hybrid structures, and advanced fabrication techniques. Given the rapid changes in the field since 2018, a new review that captures this progress, clarifies how performance demands vary by application, and differentiates between material properties and fabrication strategies is needed. This perspective aims to capture these recent advances and emphasize new strategies for patterning T/C wires and films, with a particular focus on how different chemistries and fabrication techniques balance the application-specific needs in conductivity, transparency, mechanical flexibility, and scalability required for diverse applications. This perspective highlights the intrinsic chemical properties of leading T/C materials, examines scalable and precise fabrication methods for wire and film patterning, and highlights how the combination of cutting-edge chemistries and fabrication strategies can meet application specific requirements in transparency, conductivity, and flexibility.

## History of T/C Development

### First T/C Materials

The search for T/C materials has been ongoing for over a century (Figure 4). Early efforts began with metallic meshes and thin films, which, although conductive, often lacked the necessary transparency and flexibility for advanced optoelectronic applications. A significant milestone was the development of the first T/C films in 1907 by Karl Bädeker, who discovered that Na-doped CdO exhibited both electrical conductivity and optical transparency, a combination that laid the foundation for modern T/C materials.^43^ In his work, Bädeker prepared thin films of metal compounds via sputtering onto substrates like glass or mica, followed by oxidation, iodization, or sulfurization.^44^ He used gravimetric analysis, a method that involves measuring mass, to determine film thickness and a Wheatstone bridge to measure electrical conductivity, marking the first systematic approach to characterizing T/C films.

**Figure 4:**
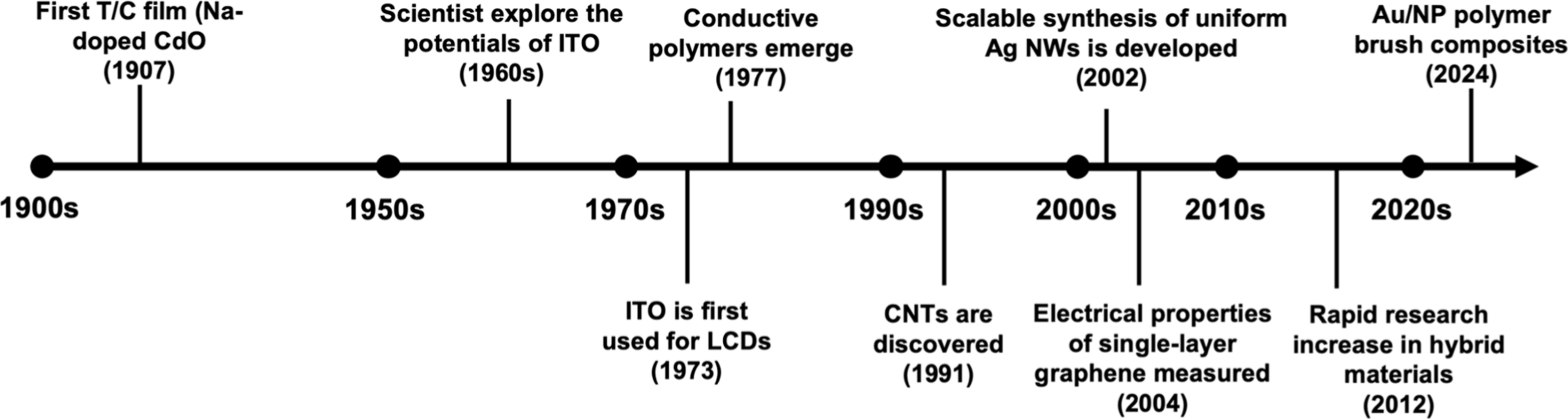
Evolution of transparent/conductive materials.

### Metal Oxides

Transparent conductive oxides, commonly referred to as metal oxides in the context of T/C materials, represent one of the most widely used classes of transparent conductors. These compounds are typically wide bandgap semiconductors (bandgaps >3 eV) that combine high optical transparency in the visible range with sufficient free-carrier density to enable metallic-like electrical conductivity.^45^ Their performance can be tailored by altering carrier concentrations through controlled stoichiometry, doping, and oxygen vacancy engineering.^46^ As a result, transparent conductive oxides can achieve transmittance values exceeding 80% while maintaining sheet resistances in the 1–100 Ω·sq^–1^ range, depending on deposition and doping conditions.^46,47^ Because of their optical clarity, electrical tunability, and compatibility with thin-film processing, metal oxides have become the dominant choice for T/C layers in applications such as flat-panel displays, touchscreens, solar cells, smart windows, and LEDs. Prototypical examples include indium tin oxide (ITO), fluorine-doped tin oxide (FTO), and aluminum-doped zinc oxide (AZO), each of which offers a different balance of conductivity, cost, stability, and scalability.

### Indium Tin Oxide

The field of T/C materials was advanced considerably in the 1970s with the introduction of indium tin oxide (ITO). Initially developed for display and electronics applications by research groups and companies in Japan and the U.S., ITO was rapidly integrated into liquid crystal displays (LCDs) and other optoelectronic devices.^48,49^ Since its discovery, ITO has been the most widely used material for preparing T/C patterns because of its balance of good optical transparency and high electrical conductivity^48,50^ as well as the well-established methods for its patterning. ITO has properties that are well suited for many optoelectronic materials that require T/C patterns. ITO has >90% transmittance in the visible spectrum (400–800 nm) and, depending upon deposition conditions, typically possesses a sheet resistance <10 Ω·sq^–1^,^51^ making it ideal for applications that require efficient electrical transport without compromising visual clarity such as touchscreen displays and thin film solar cells. Also, its Φ, ∼4.7–5.2 eV, is well-suited for charge injection and extraction in optoelectronic devices, like Si-based solar cells, where the valence band maximum (VBM) of crystalline Si lies at ∼5.17 eV and the conduction band minimum (CBM) at ∼4.05 eV relative to the vacuum level.^52^ This band alignment enables ITO to serve as a versatile transparent contact in Si solar cells. When its Φ (∼4.7–5.2 eV) is aligned with the Si valence band maximum (∼5.2 eV relative to vacuum), holes can be extracted efficiently, allowing ITO to operate as a hole-collecting anode. Conversely, when its effective work function is shifted closer to the conduction band minimum of Si (∼4.05 eV), often through interfacial layers or surface treatments, electron extraction becomes favorable and ITO functions as an electron-collecting cathode. In both cases, the close alignment between ITO’s Φ and the relevant Si band edge minimizes interfacial energy barriers, making carrier injection or extraction thermodynamically favorable. In comparison, other materials such as Au possess a higher and relatively stable Φ (∼5.1–5.7 eV),^53^ which aligns closely with the Si VBM and therefore favors hole collection. Ag, by contrast, has a lower Φ (∼4.2–4.7 eV),^54^ positioning it closer to the Si CBM and making it more suitable as an electron-collecting contact. Unlike ITO, however, the work functions of Au and Ag are not as easily tunable, which limits their versatility as dual-function transparent electrodes.

Another advantage that has facilitated widespread adoption of ITO is that methods for patterning ITO have been integrated into conventional microfabrication processes. ITO films are typically deposited using physical vapor deposition (PVD) techniques, such as magnetron sputtering^55^ or electron beam evaporation,^56^ which enable precise control over film thickness and uniformity^57^ using widely available instrumentation. By evaporating through a mask, high-quality, production-suitable ITO patterns for large-area electronics can be made. This process is currently used for the manufacture of touchscreen displays, flat-panel televisions, and photovoltaic modules, where uniformity, scalability, and optical clarity are critical for device performance.

Despite its widespread use, ITO has several limitations that hinder its applicability in emerging technologies, and these limitations motivate the search for alternatives. Its intrinsic brittleness makes it unsuitable for flexible, stretchable, or foldable devices. ITO films tend to crack or delaminate under mechanical deformation, limiting their use in wearables,^7^ biomedical sensors,^20^ and flexible displays.^26–28^ As the global demand for T/C materials continues to grow across sectors like solar energy, advanced displays, and printed electronics, the high cost of ITO production has become economically unattractive for large-scale and disposable applications,^5,58^ such as rural photovoltaic installations or low-cost medical diagnostics.^10,26^ Moreover, ITO’s electrical performance is insufficient for certain high demand applications, such as transparent antennas, electromagnetic interference shielding, and high-performance sensors, where low sheet resistance (<10 Ω·sq^–1^) is required.^59^ While ITO films can exhibit root-mean-square (RMS) roughness values below 1 nm,^60^ which are suitable for high-performance multilayer optoelectronics, many deposition methods such as plasma enhanced chemical vapor deposition (PECVD) or low-cost PVD^61^ can yield rougher surfaces (>3 nm),^62^ which limits their use in applications requiring ultraflat interfaces. ITO films exhibit a trade-off between transparency and conductivity:^63^ increasing oxygen vacancies or Sn doping enhances conductivity but also increases free-carrier absorption, lowering optical transmittance.^64,65^ Thus, optimizing ITO requires balancing stoichiometry and dopant concentration to achieve high conductivity without sacrificing visible transparency.

These shortcomings continue to drive the search for alternative materials that offer improved conductivity, flexibility, and morphological control. Some efforts include modifying ITO, by for example doping ITO with Zn, Mo, or W, to improve conductivity and tune optical properties, or to create flexible ITO films by reducing the film thickness or depositing ITO on ultrathin polymer supports.^66^ While these approaches increase the material’s flexibility, these alterations often compromise electrical performance, which further degrades following repeated deformation. Additionally, low-temperature deposition methods, including sputtering^67^ or solution-based^35^ techniques that are compatible with polymer substrates, have been explored to integrate ITO into flexible devices. However, these methods typically yield films with higher *R*_s_, reduced uniformity, or limited adhesion, particularly under thermal and mechanical stress.^36^ ITO remains the most commercially scaled transparent conductor, sustaining global display and solar cell markets, where its combination of high conductivity and optical transparency is ideal. However, its reliance on indium and vacuum-based deposition methods presents challenges. As a result, research efforts have increasingly shifted toward identifying alternative materials such as conductive polymers, metal nanostructures that can provide comparable transparency and conductivity while offering great flexibility, lower cost, or compatibility with solution-based and roll-to-roll processing.

### Other Metal Oxides

Other transparent conductive oxides such as FTO and AZO have attracted attention for their chemical stability, lower cost, and In-free composition. FTO is widely used in applications such as dye-sensitized solar cells because of its chemical stability under corrosive electrolyte conditions and relatively low cost compared to ITO.^68,69^ FTO films typically exhibit sheet resistance values of ∼10–20 Ω·sq^–1^ with >80% optical transmittance,^70,71^ though they generally have higher surface roughness (>3 nm RMS), which can limit their use in multilayer optoelectronic devices. AZO has attracted interest as an indium-free alternative, offering comparable optical transmittance (>80%) and sheet resistances in the range of 10–50 Ω·sq^–1^ depending on deposition method.^72,73^ AZO films are compatible with low-temperature and solution-based deposition, making them attractive for flexible electronics; however, their long-term environmental stability is considered to be lower than ITO, as ZnO is prone to degradation under moisture and UV exposure.^74^ Collectively, FTO and AZO highlight the potential for transparent conductive oxides to balance cost, stability, and performance for different device platforms.

### Alternative Materials

The alternatives to ITO fall into several classes: metallic nanostructures (nanowires (NWs) and nanoparticles (NPs)), conductive carbon allotropes (graphene and carbon nanotubes (CNTs)), and conductive polymers.^75,76^ Each class offers unique advantages depending on the target application and performance priorities. When selecting a T/C material, key considerations include conductivity and transparency and also mechanical durability, environmental stability, ease of patterning, Φ, and cost effectiveness. Metallic nanostructures, such as Ag NWs, provide exceptional electrical conductivity and are well suited for flexible and stretchable devices. Carbon based materials offer mechanical resilience, chemical stability, and atomic thinness, making them attractive for lightweight, flexible electronics. Conductive polymers stand out for their ease of processing, tunable properties, and compatibility with low temperature, solution-based fabrication techniques. The following sections discuss these alternatives in greater detail.

### Perforated Films

Another class of transparent conductors are perforated metallic films, in which continuous metal layers are patterned into periodic meshes or grids containing subwavelength apertures. These perforations allow visible light to pass through while maintaining metallic conduction pathways across the film.^77^ Unlike nanowire or nanoparticle networks, perforated films provide uniform, continuous conduction with sheet resistance values bellow 50 Ω·sq^–1^, while sustaining optical transmittance above 85%, depending on the aperture geometry and film thickness.^78^ Their planar morphology also reduces the surface roughness that often complicates the integration of nanowire or nanoparticle-based electrodes into multilayer devices.

Perforated films are commonly fabricated using lithographic methods such as electron-beam lithography, nanoimprint lithography, or interference lithography, followed by metal deposition and lift-off.^79^ Recent advances in nanoimprint and roll-to-roll processing have enabled larger-area fabrication, opening the possibility of scalable manufacturing.^80^ Beyond transparency and conductivity, the geometry of the perforations can be tailored to impart unique optical characteristics, such as plasmonic resonances or selective transmission, which can be exploited in specialized optoelectronic or photonic devices.^81^ Perforated metal films have found applications in transparent antennas, plasmonic circuitry, and optoelectronic devices requiring electrodes with both mechanical robustness and tailored optical properties.^82,83^ However, their reliance on nanoscale lithography currently limits widespread commercialization because of the complexity and cost of these fabrication methods, restricting use mainly to applications where performance advantages outweigh processing challenges.^19^

### T/C Nanowires

NWs are one-dimensional nanostructures with diameters typically <100 nm and lengths up to several microns.^84^ Their high aspect ratio, combined with quantum and surface effects, imparts unique optical, electrical, and mechanical properties, making them excellent candidates for T/C films. NWs can be broadly classified into metallic NWs (Ag NWs, Cu NWs, Au NWs) (Figure 5A), semiconducting NWs (Si NWs, ZnO NWs), and dielectric NWs (TiO_2_ NWs, SiO_2_ NWs), each offering distinct advantages depending on the application.^85–87^

**Figure 5:**
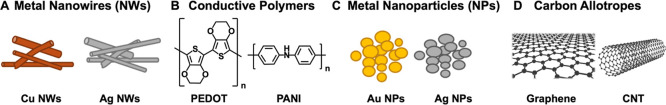
Alternative materials for T/C films. (A) Ag NWs and Cu NWs. (B) Structures of conductive polymers PEDOT and PANI. (C) Au (yellow) and Ag (gray) NPs. (D) A layer of graphene and a CNT.

Ag NWs, the most widely studied metallic NW system,^88–91^ combine excellent electrical conductivity (*R*_s_ typically 10–30 Ω·sq^–1^) with mechanical flexibility, making them suitable for flexible displays, stretchable sensors, and wearable electronics. In addition to conductivity and flexibility, their Φ, typically ∼ 4.1–4.9 eV for polyvinylpyrrolidone stabilized Ag NWs, plays a critical role in determining device compatibility.^92^ For example, in stretchable organic thin-film transistors employing p-type (hole-transporting) semiconductors, the relatively lower work function of Ag NWs often mismatches the HOMO level of the organic semiconductor (generally deeper than 5.0 eV), creating an injection barrier for holes and limiting device performance.^93^ This makes Ag NWs naturally better suited for electron collecting electrodes in photovoltaics, photodetectors, and certain display architectures, while surface treatments or interlayers can be used to raise the work function and enable efficient hole injection in OLEDs and other p-type devices.^94,95^ Ag NWs’ exceptional conductivity and mechanical flexibility have made them a promising alternative to brittle oxides like ITO, especially for applications requiring stretchability or bendability. Ag NWs are commonly synthesized via solution-based methods, such as the polyol process,^88,96–98^ which involves the reduction of AgNO_3_ in ethylene glycol with polyvinylpyrrolidone (PVP) as a capping agent. Other methods include hydrothermal syntheses^99^ and template assisted growth.^100^ Ag NW networks are typically deposited via spray coating, spin coating, or inkjet printing.^101–103^ Recent advances in Ag NW-based T/C films have enabled performance that match, and in some cases exceed, those of standard commercial ITO, which typically exhibits a sheet resistance of ∼ 30 Ω·sq^–1^ at ∼ 85% optical transmittance. For instance, high-speed photonic curing, an annealing technique that rapidly sinters Ag NW networks using intense pulsed light, has produced *R*_s_ as low as 9.8 Ω·sq^–1^ with >90% transparency,^104^ outperforming typical ITO electrodes by a factor of 2.6–2.7. These improvements are compatible with low-temperature processing and scalable R2R manufacturing, greatly enhancing their potential for widespread deployment in printed and flexible optoelectronic devices.

Further enhancements in conductivity and mechanical stability of Ag NW T/C materials have been achieved by tailoring the interfaces between conductive layers and adjacent materials—for example, by introducing interfacial adhesion layers, or incorporating buffer layers to reduce contact resistance and improve charge transport. One study demonstrated that Ag NWs deposited via spray transfer, a process in which a suspension of nanowires is atomized and sprayed onto a substrate to form a film, when combined with monodisperse silica nanoparticles (SiO_2_ NPs) and a polyurethane acrylate coating, can form semiembedded T/C films with excellent uniformity, high optical transmittance (93.9%) in the visible range, and low sheet resistance (13.4 Ω·sq^–1^).^105^ This approach overcomes common limitations of Ag NW films, such as poor adhesion and wire-to-wire contact resistance, by improving mechanical robustness, environmental stability, and conductivity uniformity without sacrificing flexibility.^90^ From a device integration perspective, incorporating Ag NWs into layered architectures can be complicated by surface roughness, poor adhesion to substrates, and rough film morphology. These factors can interfere with layer-to-layer contact in multilayer devices, such as OLEDs or capacitive touchscreens, and as a consequence, fabrication of such devices using Ag NWs may require additional planarization steps or interface engineering to ensure reliable performance. Addressing both stability and integration issues remains critical for advancing Ag NWs from promising materials to fully commercialized components in optoelectronic systems. While Ag NW-based electrodes are compatible with solution processing and R2R manufacturing, scalability challenges persist, particularly in achieving uniform large area coatings, controlling wire alignment and density, and minimizing junction resistance without compromising transparency or flexibility.^106^

### Conductive Polymers

Conductive polymers are organic polymers that exhibit electrical conductivity arising from the conjugated π-electron systems along their backbones,^107^ and have been explored in the context of T/C materials for optoelectronic devices, such as flexible displays, sensors, and photovoltaics.^108^ These macromolecules can transport charge carriers through delocalized π-orbital networks, and while they are intrinsically semiconducting, their electrical conductivity is significantly enhanced upon doping with appropriate oxidizing or reducing agents.^107^ Beyond increasing conductivity, dopants also modify the polymer’s work function, enabling energy level alignment with adjacent layers for efficient charge injection or extraction in devices.^109,110^ For example, p-doping poly­(3,4-ethylenedioxythiophene) (PEDOT) can raise Φ to ∼5.0–5.2 eV, making it suitable as a hole-injection layer in OLEDs,^111,112^ while n-doping polymers such as naphthalene diimide derivatives can lower Φ to ∼4.0–4.3 eV for electron transport applications.^113–115^ The transport properties of conductive polymers are highly dependent on doping levels and processing conditions. Increased doping improves conductivity but can also reduce transparency because of increased absorption in the visible range. Similarly, higher molecular ordering and crystallinity can improve carrier mobility but may scatter light, thereby reducing transparency of the films.^116^ While there are many known conductive polymers, only a limited subset are viable for T/C applications, as many either absorb strongly in the visible region or suffer from poor solubility and processability or have work functions that are poorly matched to the needs of most T/C device architectures. As such, a careful balance must be achieved between electrical performance and optical transmittance when working with conductive polymers. Optimizing this trade off involves tailoring the polymer formulation, processing method, and post-treatment techniques to maximize charge transport without compromising transmittance. For device fabrication, conductive polymers can be patterned using inkjet printing,^117^ spray coating,^118^ spin coating,^119^ and photolithography,^120,121^ and they are particularly attractive for applications requiring conformal or flexible form geometries on substrates that include plastics and textiles. The integration of conductive polymers into devices through printing and lithographic techniques offers a versatile and customizable approach to fabricating T/C wires and patterns. These techniques enable precise patterning on a wide range of substrates, from rigid materials, like glass, to flexible options such as polyethylene terephthalate (PET) and polyimide. This adaptability is particularly advantageous for technologies requiring conformal surfaces or bendable components, such as wearable sensors and flexible displays. Ensuring scalability and consistent performance requires addressing challenges such as substrate compatibility, surface preparation, and potential deformation during processing.

The most common conductive polymer for T/C applications is PEDOT^122,123^ (Figure 5B), particularly in its doped form PEDOT/PSS, a complex comprising positively charged PEDOT chains and the polyanion poly­(styrenesulfonate) (PSS), which serves as both a charge balancing counterion and a dispersant to improve water solubility. PEDOT/PSS is the material of choice for many T/C applications because of its high visible light transmittance (typically >80%), excellent flexibility, and tunable *R*_s_–ranging from ∼10–500 Ω·sq^–1^ depending on formulation and postdeposition processing.^124–126^ These properties make it attractive for flexible displays,^127^ OLEDs,^128^ touchscreens,^124^ organic solar cells,^129^ electrochromic devices,^130^ and wearable sensors,^131^ where mechanical compliance and solution processability are required. These materials are typically synthesized via chemical or electrochemical polymerization and are compatible with low-temperature, solution-based fabrication methods, such as inkjet printing, spin coating, spray coating, and doctor blading, enabling deposition on a wide range of flexible substrates including PET^132^ and textiles.^133^ Major research in this field focuses on improving the conductivity/transparency balance,^134^ enhancing environmental stability,^135^ optimizing mechanical durability under strain,^136^ and developing scalable patterning methods that are compatible with R2R processing and multimaterial integration.^132^

In addition to PEDOT/PSS, other conductive polymers such as polyaniline (PANI) and polypyrrole (PPy) (Figure 5B) have been explored for T/C applications, particularly in contexts where flexibility, multifunctionality, and optical clarity are critical. PANI has demonstrated promise in enhancing the electrical performance of transparent, flexible sensors. For example, in a trilayer structure combining graphene and PANI on a polydimethylsiloxane (PDMS) substrate, the inclusion of a PANI interlayer significantly improved conductivity.^137^ The sheet resistance of a first-layer graphene film was measured at 357 Ω·sq^–1^, which decreased to 277 Ω·sq^–1^ with the addition of a PANI layer. The final graphene–PANI–graphene (G–P–G) configuration achieved a sheet resistance of 84.3 Ω·sq^–1^, representing a 4-fold improvement over pristine graphene and over an order-of-magnitude lower resistance than standalone PANI (957 Ω·sq^–1^). This G–P–G structure also maintained ∼90% optical transmittance and exhibited robust mechanical compliance and signal stability under strain, enabling reliable detection of human motion, respiration, and pulse. This G–P–G configuration surpassed the performance of single layer graphene structures and also exhibited robust mechanical compliance and reliable signal transduction under strain, enabling diverse sensing functions such as human motion, respiration, and pulse detection. This highlights PANI’s utility in mechanically dynamic, wearable sensing platforms.

PPy, on the other hand, has been integrated with nanostructured materials, like MXenes, to create hybrid films for transparent heater applications.^138^ When coated onto polycarbonate substrates as part of a MXene/PPy composite, PPy enhanced the photothermal conversion efficiency and electrical conductivity of the film. The resulting heater exhibited a surface resistance of 413 Ω·sq^–1^, transmittance of 52%, and stable heating, reaching 108 °C at 24 V.^138^ Furthermore, it remained stable over seven months, underscoring PPy’s potential in outdoor or biomedical settings, such as light-triggered thermal therapy films for skin. These studies collectively demonstrate that, although less commonly used than PEDOT/PSS, both PANI and PPy can be tailored for specific flexible T/C electronic devices where dual functionality, such as sensing and thermal control, is desired.

### Conductive Carbon Allotropes

Conductive carbon-allotropes, including graphene and fullerenes—particularly CNTs (Figure 5D)—have emerged as promising candidates for T/C applications as a result of their exceptional flexibility, chemical stability, and electrical conductivity.^2,26,139,140^ Their atomic-level thinness and mechanical resilience make them especially attractive for applications that demand stretchable, foldable, or wearable designs. Graphene, a two-dimensional sheet of sp^2^-bonded carbon atoms,^141^ exhibits high carrier mobility and transparency.^142^ While CNTs, by contrast, are cylindrical nanostructures whose structure is that of single or multiple graphene sheets rolled into tubes, offer conduction pathways with tunable band gaps based on their chirality.^143,144^ Their Φ are typically around 4.95 eV for multiwalled CNTs and 5.05 eV for single-walled CNTs, values that make them suitable for applications requiring efficient hole injection or extraction, depending on the device architecture.^145^ These all-sp^2^ carbon allotropes have been explored extensively for use in transparent electrodes,^146^ flexible displays,^147^ electronic skins,^148–150^ and sensors.^151^ Among the most interesting examples of using conductive carbon allotropes to make T/C devices involves integrating CNT networks and graphene into circuits and sensors.^149,152^ In one particularly noteworthy example, the Bao group designed intrinsically stretchable CNT thin-film transistors with megahertz operational speeds and introduced a circular channel architecture that dramatically reduced strain-induced current variation from over 50% to just a few percent.^153^ In a separate study, they developed solution-processable CNT/conductive polymer composite films and fibers using an in situ polymerization strategy, in which a conducting polymer, such as PEDOT or PANI, was polymerized directly within a prealigned CNT network. This approach enhanced interfacial connectivity and provided mechanical support, resulting in a two order-of-magnitude increase in electrical conductivity. The resulting films achieved conductivities as high as 3300 S/cm, which corresponds to a sheet resistance of approximately 3 Ω·sq^–1^ for a 1 μm-thick film.^154^ Further pushing the boundaries of stretchable T/C materials, the group introduced a multilayer graphene/graphene nanoscroll (MGG) structure into stretchable transistor electrodes, which preserved over 65% of their initial conductivity under 100% strain, substantially outperforming monolayer graphene, which typically fails at 5% strain. These MGG-based electrodes enabled the fabrication of all-carbon, stretchable transistors with >90% optical transparency and stable operation up to 120% strain.^155^ The incorporation of graphene scrolls created bridging pathways that maintained network conductivity, even as fractures formed under tensile deformation. These properties mark a critical step toward commercial deployment in wearable optoelectronics and implantable biosystems, where mechanical durability, electrical stability, and optical clarity are essential.

Despite their promise, carbon allotropes face persistent challenges that have limited their widespread commercialization. Graphene’s scalability remains a hurdle because of continuing difficulties in producing large area, defect-free films with consistent electrical performance.^156,157^ Similarly, CNT-based films often suffer from issues like tube aggregation, inconsistent alignment, difficulties in producing homochiral tubes, and high junction resistance.^158^ Moreover, both materials—graphene and CNTs—currently have high production costs and complex transfer or coating processes, complicating their integration with standard device fabrication workflows.^157,158^ Addressing these challenges is essential for enabling carbon-based T/C materials that are high-performing and also scalable and environmentally stable.

### Nanoparticle T/C Materials

Metallic NPs^159^ (Figure 5C), especially AuNPs,^160,161^ have gained attention as promising components in T/C materials as a consequence of their tunable optical and electronic properties, nanoscale dimensions, and potential for low temperature processing. An advantage of NPs is that they can be incorporated into polymer matrices to create composite materials with desirable optical and electrical properties.^27,162^ For example, Au NPs incorporated into conductive polymers such as PEDOT, have demonstrated significant improvements in film conductivity. One study showed that embedding 12 nm Au NPs into PEDOT increased conductivity by over 7-fold compared to pristine PEDOT films (800 Ω·sq^–1^), achieving a sheet resistance of 85 Ω·sq^–1^ at 85% transmittance.^161^ This enhancement in conductivity arises from charge-transfer interactions and hopping mechanisms between PEDOT and Au NPs. The hybridization process interconnects PEDOT chains with Au NPs, enabling electron transfer in both directions and effectively pinning the Fermi level inside the valence band, which increases the doping level and carrier density. Larger Au NP sizes further improve conductivity by providing greater surface area for interfacial charge transfer, consistent with the observed 7-fold increase in conductivity. The Φ of these Au NP/PEDOT hybrid films, measured by photoelectron spectroscopy, was in the range of 4.8–5.1 eV, which is comparable to that of indium tin oxide (ITO), making them suitable for applications requiring efficient hole injection or extraction.^161^ Another major advantage of NP-based T/C layers is their compatibility with patterning techniques that include inkjet printing^163^ and photolithography,^164,165^ which stems from their ability to be dispersed in solvents or formulated into stable colloidal inks. This solubility enables uniform deposition onto substrates through solution-based methods, allowing for precise control over NP distribution and feature geometry. For instance, Au NP inks formulated using polymeric capping agents and optimized solvent compositions have been used to produce stable, clog-free colloids with average particle sizes of ∼5.6 nm.^166^ Inkjet-printed films from these inks, followed by thermal sintering, achieved sheet resistance values below 2 Ω·sq^–1^.^166^ In inkjet printing, the ability to tune viscosity and surface tension through solvent choice is critical for achieving consistent droplet formation and resolution^167^ and in this study they were able to highlight the ability to tailor these properties using Au NP inks.

Similarly, in photolithography, well dispersed NPs can be blended into photoresist formulations^168^ or selectively deposited via lift-off techniques.^169^ In addition, in situ incubation of metal NPs within polymer matrices, where metal salts are incorporated into the film and subsequently reduced thermally, chemically, or photochemically, either during or after patterning, can lead to enhanced interactions between the NP and the polymer matrix, reduced aggregation, and provides a tunable conductivity by controlling NP growth and distribution at the nanoscale.^170^ When deposited appropriately, metal NPs can exhibit high electrical conductivity, strong surface plasmon resonance, and excellent chemical stability, making them suitable for integration into sensitive and long-lasting electronic components.^171^ While surface plasmon resonances in metal nanostructures can enhance near-field effects and conductivity, they also lead to increased optical absorption at resonance wavelengths, reducing transparency.^172^ Thus, careful control over nanoparticle size, shape, and distribution is necessary to balance plasmonic conductivity gains with optical losses. Despite these promising developments, key challenges remain. Achieving long-term stability, scalability, and reproducibility of NP-based T/C films is still a critical hurdle. Additionally, balancing conductivity with optical clarity while minimizing surface roughness and NP aggregation requires careful formulation and process optimization.

### Titanium Nitride

Titanium nitride (TiN) has recently emerged as a promising candidate among metallic transparent conductors.^173^ Ultrathin TiN films combine good electrical conductivity with favorable optical properties, especially in the visible and near-infrared range, while also showing enhanced thermal and chemical stability compared to metals. For example, ultrathin TiN epitaxial films (2–10 nm thick) grown by nitrogen plasma-assisted molecular beam epitaxy (MBE) demonstrate sheet resistances under 100 Ω·sq^–1^, with optical transmittance exceeding 75% in the visible spectrum, depending on film thickness and growth conditions.^174^ TiN also exhibits plasmonic behavior: its permittivity becomes negative above certain wavelengths, allowing localized surface plasmon resonances. Compared to noble metals, TiN retains more stable transparency and conductivity at elevated temperatures and under harsh conditions, making it suitable for applications where robustness is critical.^175^

Despite these advantages, the trade-offs in working with TiN are significant: as TiN becomes thinner, scattering at grain boundaries, surface roughness, and film continuity greatly influence both optical loss and sheet resistance.^176,177^ Achieving smooth, continuous ultrathin films requires careful control of deposition, substrate selection, and postdeposition treatments. Overall, TiN represents a compelling option for transparent conductors, especially in applications demanding high temperature stability, chemical resilience, or harsh-environment operation, although in many cases it does not yet match the low sheet resistance/high transparency achieved by optimized ITO or Ag-based transparent films.

### Fabrication Methods for Transparent Conductive Wires and Patterns

While intrinsic material properties and processing conditions to a large extent govern electrical, optical, and mechanical performance of the T/C layers, the ability to precisely pattern T/C materials is what enables their successful integration into device architectures, while also affecting optoelectronic and material properties. Patterning dictates the location of the conductive channels, how the material interacts with light, interfaces with other device layers, and contributes to the size, shape and flexibility. In next-generation optoelectronics, particularly in touchscreens, photovoltaics, and wearable sensors, fine resolution patterning is essential to achieve application specific performance metrics in feature density, conductivity, transparency, and mechanical compliance.

Patterning techniques can be broadly categorized into five major classes (Figure 6) based on the deposition mechanism and equipment used: solution-based processing, vacuum-based deposition, R2R manufacturing, photolithography, and soft lithography. Each class is distinguished by key factors such as substrate compatibility, patterning resolution, processing temperature, material throughput, and scalability. For instance, solution-based methods are typically low-cost and compatible with flexible substrates, while vacuum-based approaches offer superior film quality but typically require cleanroom conditions. Lithographic techniques offer the highest resolution and alignment precision, and R2R processing excels in scalability and continuous fabrication for large-area applications.

**Figure 6:**
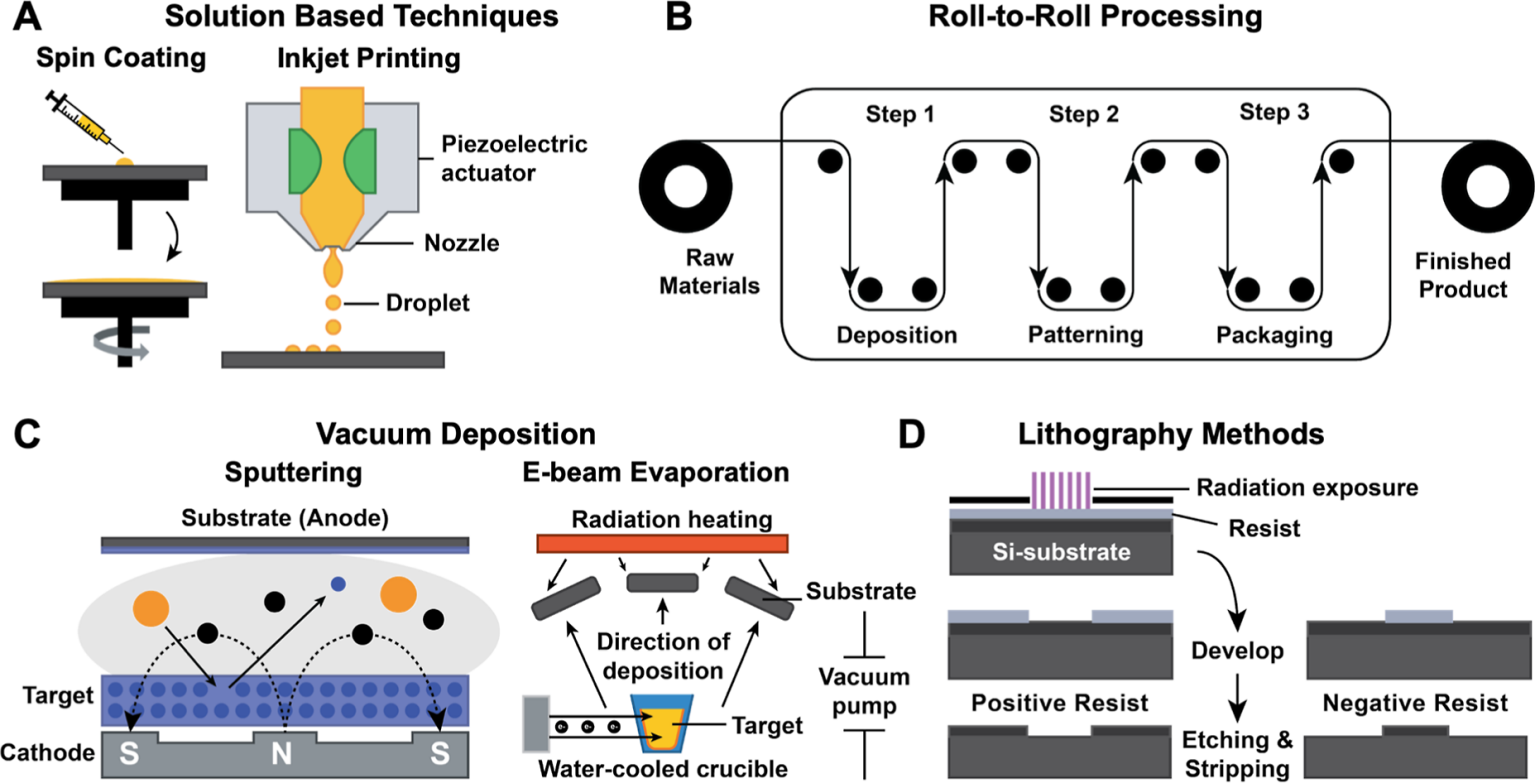
Fabrication methods for transparent conductive patterns. (A) Solution based techniques: spin coating. (Adapted from ref 182). Inkjet printing. (Adapted from ref 184). (B) Step by step of R2R fabrication. (Adapted from ref 197). (C) Vacuum deposition techniques: sputtering (orange circles represent Ar^+^ and black circles represent e−), E-beam evaporation. (Adapted from ref 190.). (D) Lithography methods: photolithography (Adapted from ref 209).

### Solution-Based Deposition of T/C Films

Solution based fabrication techniques offer a versatile, low-cost route for depositing and patterning T/C materials, particularly on flexible or temperature sensitive substrates such as PET, polyimide, and textiles.^178^ These methods rely on the formulation of T/C materials, such as Ag NWs,^101,179^ CNTs,^51^ graphene,^157^ and conductive polymers,^119,123^ into printable or coatable inks that can be applied onto. Among the most commonly used techniques is spin coating,^180^ which enables the deposition of uniform thin films by centrifugal spreading of a solution (Figure 6A). It is widely used for conductive polymer formulations^123^ and sol gel precursors to ITO,^181,182^ though it is typically limited to flat substrates and small areas. To obtain patterns from spin-coated films, subtractive methods such as photolithography or soft lithography are often employed postdeposition, where undesired regions are removed through etching or lift-off techniques. Spray coating provides more uniform coverage and can be used over larger, nonplanar surfaces, making it suitable for wearable and textile-based electronics.^179^ Patterning after spray deposition typically requires shadow masks during spraying or postprocessing steps like laser ablation or plasma etching to define features. Inkjet printing stands out for its ability to digitally pattern T/C inks without the need for physical masks or stencils^101^ (Figure 6A). This technique enables additive manufacturing with high material efficiency and spatial resolution, making it ideal for rapid prototyping or multimaterial printing.^183^ Each of these solution-based methods offers a different balance of scalability, precision, and compatibility with flexible substrates, and the choice of postdeposition or direct patterning strategy depends on the intended device architecture and resolution requirements.

Despite their promise, solution-based approaches face several challenges. Achieving uniform film morphology can be difficult because of ink spreading or nozzle clogging in printing systems.^184,185^ Additionally, many materials require postdeposition processing, such as thermal annealing, photonic sintering, or chemical treatments, to enhance conductivity, adhesion, and film uniformity.^186^ While inkjet printing enables direct pattern formation during deposition, other techniques like spin and spray coating typically require postpatterning steps such as photolithography, soft lithography, or selective etching to define features.^180^ These additional steps can add complexity and may limit resolution. Although the spatial resolution of solution-based methods still falls short of photolithography, ongoing advances in ink formulation, surface energy control, and printing hardware continue to improve their pattern fidelity and reproducibility.^187^ Overall, solution-based techniques remain a leading strategy for fabricating flexible, lightweight, and large-area T/C patterns at low cost, especially when combined with optimized postpatterning processes.

### Vacuum Deposition of T/C Films

Vacuum based deposition techniques are among the most well-established methods for fabricating T/C films, particularly for materials like ITO, Al-doped ZnO (AZO), and thin metal layers (Ag, Au).^36,91,106^ These methods include sputtering^188,189^ (Figure 6C), thermal evaporation, electron beam evaporation^190,191^ (Figure 6C), and PECVD,^190^ each offering control over film thickness, uniformity, and stoichiometry sputtering, especially radio frequency magnetron sputtering, is widely used for depositing ITO and other oxide-based conductors.^192^ This technique involves bombarding a target material with high-energy ions, ejecting atoms that then condense onto a substrate to form a thin film. It allows for precise tuning of film composition and thickness by controlling parameters such as gas pressure, power input, and target–substrate distance. Moreover, sputtering systems can accommodate large substrate sizes and operate continuously in industrial-scale vacuum chambers, enabling uniform film deposition over areas exceeding square meters. This scalability, combined with excellent film quality and compatibility with existing manufacturing lines, has made sputtering the dominant method in commercial display and photovoltaic production.^193^ Thermal and electron beam evaporation, while less common for ITO, are effective for depositing ultrathin metallic layers in multilayer architectures, such as Ag interlayers in oxide-metal-oxide structures.^190,194^

Despite their precision, vacuum-based methods come with notable trade-offs. The need for high vacuum conditions and specialized equipment results in high capital and operating costs, limiting their accessibility for low cost or flexible electronics. Furthermore, many of these processes require elevated substrate temperatures (typically >200 °C), which are incompatible with most polymer substrates.^195^ Techniques like PECVD, while useful for depositing conductive and barrier layers at lower temperatures, can introduce surface roughness and other morphologies that impair film flatness, which is a limitation for optical applications requiring atomically smooth interfaces.^196^ In summary, vacuum-based deposition is the leading technique for high-quality, high-performance T/C films, especially in rigid electronics. However, it can be incompatible with certain flexible substrates, requires an additional element to create patterns, and cost limitations have motivated the development of alternative processing strategies better suited to emerging applications.

### Roll-To-Roll Printing of T/C Layers

R2R printing is a high-throughput, continuous manufacturing approach designed for large area, flexible substrates, and it is especially well suited for flexible electronics involving T/C materials deposited on polymer substrates (Figure 6B).^197^ R2R processes, such as gravure printing, slot die coating, and flexographic printing, enable the deposition of T/C inks including Ag NWs, graphene, and PEDOT/PSS onto flexible substrates like PET or polyimide under ambient or low-temperature conditions.^198–201^ The advantage of R2R printing lies in its scalability and cost efficiency.^13,202^ It supports rapid production over meter scale lengths, making it ideal for commercial applications like smart windows, rollable displays, wearable sensors, and flexible photovoltaics.^199,200^ Moreover, additive R2R processes minimize material waste, and some methods can incorporate real-time quality control and multimaterial patterning within a single pass.

However, R2R printing has several limitations. Achieving high resolution features (below ∼50 μm) can be difficult, and surface tension effects, ink substrate interactions, and pattern alignment must be precisely controlled.^202,203^ Mechanical deformation, film nonuniformity, and drying artifacts may further impact conductivity and optical clarity.^204,205^ Additionally, inks must be formulated for specific R2R compatible flow, and many require postprinting sintering or annealing, which can constrain substrate selection.

Despite these challenges, R2R is one of the most promising fabrication platforms for scaling T/C technologies beyond laboratory demonstrations to widespread commercial adoption. Its compatibility with solution processing and low-cost substrates aligns with the growing demand for wearable, disposable, and large-area optoelectronic devices.

### T/C Patterns via Photolithography and Soft Lithography

Photolithography is the most widely used high-resolution patterning method in microelectronics and remains the industry standard for fabricating precise T/C wire architectures.^206^ It typically involves coating the substrate with a photoresist, exposing it to patterned UV light through a mask, and developing the resist to define fine features, followed by etching or deposition^207–209^ (Figure 6D). Photolithography enables micron to submicron scale features with excellent edge definition and alignment accuracy,^210^ which is critical for applications like OLEDs, micro-LED displays, and sensors.

A notable example is the successful patterning of conductive PEDOT/PSS films using a silver interlayer.^120^ In this approach, ∼100 nm of silver was deposited on PEDOT/PSS films and patterned through conventional photolithographic steps, followed by silver removal via etching.^120^ The resulting patterned films achieved a sheet resistance of 173.2 Ω·sq^–1^ at 91% transmittance, and were used to fabricate OLED devices that demonstrated performance comparable to those with ITO anodes. This work highlights photolithography’s capability to produce high-resolution, large-area patterned electrodes compatible with flexible substrates, while maintaining excellent electrical and optical properties.

Traditional photolithography is limited by substrate rigidity and processing complexity.^153^ It often requires multiple steps, has high equipment costs, and is generally incompatible with flexible, stretchable, or thermally sensitive substrates because of high-temperature baking and resist processing steps. To address these limitations, soft lithography has emerged as a versatile and low-cost alternative for patterning T/C materials on nonrigid substrates. Unlike photolithography, soft lithography uses elastomeric stamps or molds, typically made from PDMS, to define micro and nanoscale features through mechanical contact rather than light exposure.^211^ Techniques such as microcontact printing,^212^ replica molding,^213^ and micromolding in capillaries/capillary force lithograhy^213^ allow T/C inks, conductive polymers, or NP formulations to be patterned directly onto flexible, stretchable, or even curved surfaces without requiring high temperatures or vacuum-based processing. For example, capillary force lithography combined with simple cross-stamping has been used to create periodic dots, rings, and line patterns of poly­(ethylene oxide)/HAuCl_4_ composites.^214^ By controlling the wettability contrast between the substrate and the PDMS mold, isolated dot patterns could be formed, with their shape and size tuned by adjusting the initial film thickness. Chemical reduction of the precursor yielded ordered arrays of Au nanorings or nanodots, demonstrating the technique’s potential for fabricating patterned conductive structures on flexible substrates. Soft lithography is especially attractive for wearable sensors, bioelectronics, and flexible displays, where mechanical compliance and low-temperature compatibility are essential. Its ease of use, low cost, and compatibility with soft substrates make it an ideal complement to conventional patterning approaches, particularly in emerging device platforms that demand mechanical adaptability.

However, despite these achievements, current top-down fabrication methods, including photolithography, inkjet printing, screen printing, and soft lithography, still face critical limitations. These include challenges in high-throughput screening of new materials, complex multistep processes, limited resolution on soft or nonplanar substrates, and difficulties in achieving precise spatial control over both material composition and film thickness in a single step. Additionally, scaling up these techniques while maintaining reproducibility and performance consistency remains an ongoing concern. Notably, all of these approaches are top-down, relying on subtractive or stencil-based methods to define patterns from bulk materials. To overcome these limitations, there is increasing interest in bottom-up fabrication strategies that enable simultaneous material synthesis and patterning.

### Hypersurface Photolithography

The convergence of advanced chemistry, novel materials, and innovative lithographic techniques is driving the next wave of breakthroughs in T/C material design and patterning. By merging high-resolution patterning technologies with tunable chemistry, researchers can fabricate functional architectures with unprecedented control over spatial resolution, material composition, and device integration.

One example of this multidisciplinary strategy involves the use of hypersurface photolithography (HP) for creating multicomponent T/C patterns (Figure 7A). HP combines a digital micromirror device (DMD) to project light with surface-initiated photopolymerizations to pattern polymer brushes or functional materials with micron-scale spatial resolution and nanometer-scale control of brush height, enabling the preparation of well-defined features on a variety of substrates.^162,215–218^ A major advantage of HP is that each feature in a pattern can be prepared using different reaction conditions, so that the effects of a wide range of reaction conditions on features height and T/C properties can be rapidly screened. Recent work utilizing HP demonstrates^162^ the optimization and fabrication of T/C composite films composed of polymer brush/Au NP composites. This was accomplished by using HP to create polymer brushes containing the Au-binding monomer 2-vinylpyridine (2VP). Subsequent incubation of the resulting brush polymer patterns with Au^4+^ ions, followed by in situ reduction of the Au^4+^ ions into Au NPs resulted in T/C patterns composed of Au NPs adhered to the polymer brush patterns. The resulting patterns achieve high resolution conductive structures with optical transmittance >85% and sheet resistance <1 Ω·sq^–1^ ^162^ (Figure 7E,F). Unlike vacuum-based methods, HP operates entirely under solution conditions, does not require clean-room facilities, and is compatible with a wide range of substrates, including plastics and soft materials.

**Figure 7:**
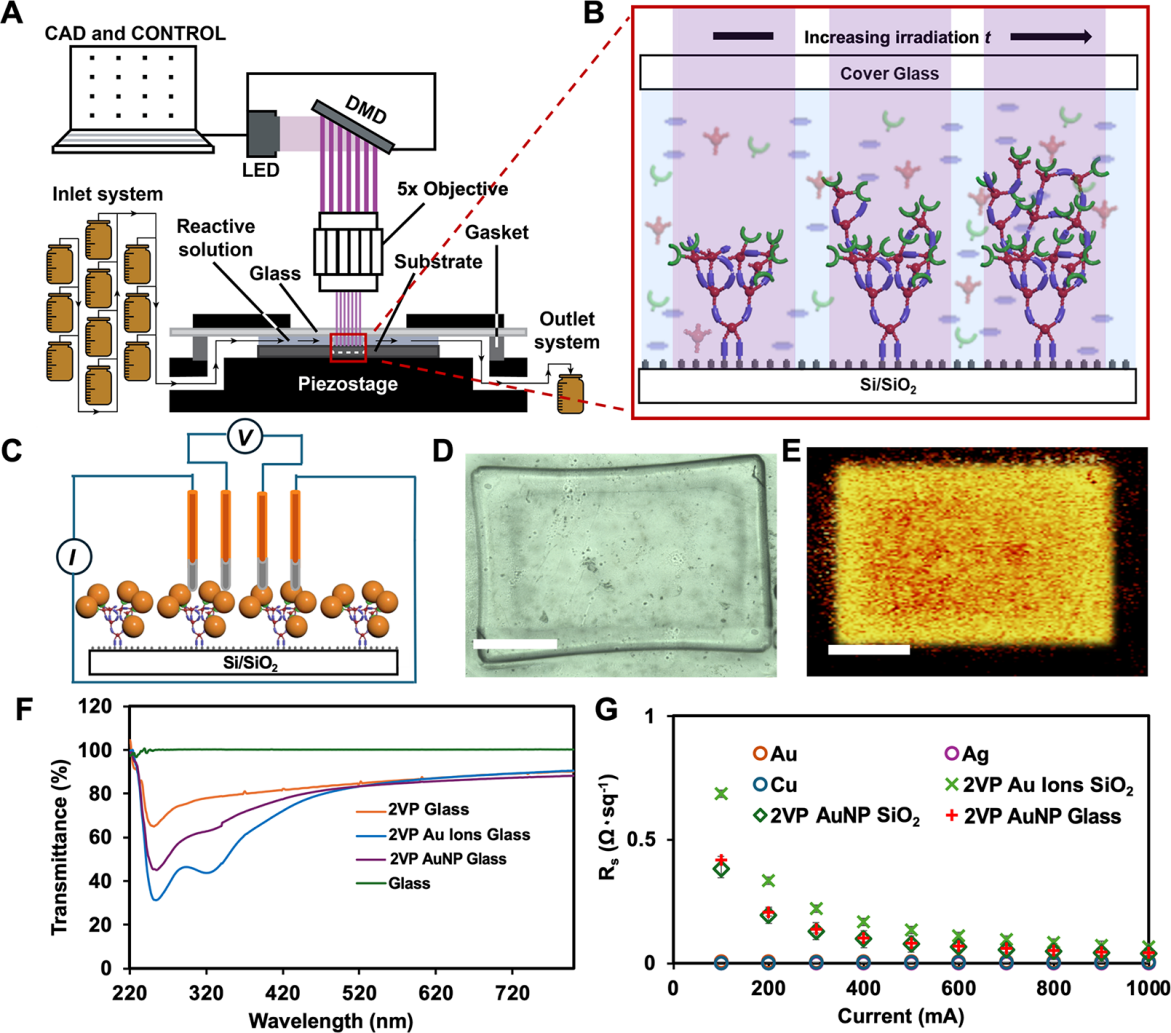
Bottom-up patterning of transparent and conductive polymer brush films via Hypersurface photolithography (HP). (A) The HP printer used for surface patterning combines a digital micromirror device (DMD), a microfluidics-enabled fluid cell, and a reactive surface. (B) Patterned polymer brushes growing from a thiol-terminated Si/SiO_2_ surface by consuming monomers in solution upon exposure to light (purple lines). As the irradiation time increases, the height of the polymer brushes increases. (C) 2VP-EGDMA-PETT polymer brushes on thiol-terminated Si/SiO_2_ surface upon reduction of Au-ions in with NaBH_4_, forming AuNPs with a 4-point probe contact for *R*_s_ measurements. (D) Optical image of 2VP-EGDMA-PETT polymer brushes bound to AuNPs prepared via in situ reduction on a thiol-functionalized glass surface passivated with maleic anhydride. Scale bar is 900 μm. (E) Raman map (λ_ex_ = 532 nm) of peaks corresponding to Au (310–350 cm^–1^) of 2VP-EGDMA-PETT polymer brushes with intercalated AuNPs. Scale bar is 1000 μm. (F) Transmittance data taken from the sample (G). (I) Sheet resistance (*R*_s_) vs current (*I*) plot of different samples measured by a 4-point probe. Figure adapted with permission from ref 132. Copyright 2025 John Wiley and Sons Inc.

The maskless patterning capabilities of HP, combined with its compatibility with advanced polymer chemistries and metal NP assembly, make it an exceptionally versatile platform for next-generation device manufacturing. Applications include biosensors and transparent circuitry, where traditional lithographic methods often fall short due to limitations in substrate flexibility, resolution, or processing temperature. Despite its advantages, HP and soft lithography methods are still under development for high-throughput industrial use. Challenges include alignment over large areas, consistency across batches, and integration with multilayer device architectures. Nevertheless, the patterning flexibility and material compatibility of HP, in combination with its ability to screen rapidly a variety of patterning conditions, offer a promising complement to traditional lithography in T/C applications or for discovering new materials or processing conditions that could then be transitioned to more high-throughput patterning techniques.

## Conclusions

T/C wires and patterns form the backbone of a wide array of modern optoelectronic technologies, from displays and solar cells to smart windows and wearable electronics. The continued evolution of these technologies has driven a parallel need for materials that deliver high transparency and low *R*_s_, appropriate Φ, mechanical flexibility, environmental stability, and scalable manufacturing compatibility. While ITO has served as the industry standard for decades, its brittleness and limited compatibility with flexible substrates have prompted widespread exploration of alternative materials and fabrication strategies. This perspective highlights the emergence of metallic NW and NPs, carbon allotropes, and conductive polymers as promising ITO alternatives. Each material class brings unique advantages and limitations in terms of chemistry, performance, integration, and scalability. To effectively implement these materials into functional devices, a diverse set of patterning techniques, including solution-based printing, vacuum deposition, R2R manufacturing, and both conventional and emerging, have been developed and refined. These fabrication methods enable the spatial control necessary for device operation and also the tuning of electrical and optical properties to meet specific performance targets.

The field of T/C materials is now entering a stage where interdisciplinary innovation is key, and innovation will be accomplished by combining cutting-edge materials with advanced lithographic techniques for next-generation T/C architectures. Lithography innovations, such as HP, demonstrate how programmable, maskless, and bottom-up fabrication strategies can produce high-performance, transparent, and flexible patterns using solution-processable materials. Looking ahead, continued progress will require integrated efforts in materials chemistry, nanostructure engineering, and scalable processing. Addressing remaining challenges, such as long-term stability, interfacial compatibility, and large-area uniformity, will be critical for translating laboratory breakthroughs into commercially viable products.

In parallel, new advances in the integration of artificial intelligence (AI) with materials science promises to fundamentally accelerate the discovery and optimization of new T/C materials. By leveraging large experimental data sets, high-throughput simulations, and machine learning models, AI can help predict structure property relationships, identify promising chemistries, and optimize synthesis conditions with unprecedented speed. This shift from trial-and-error discovery to intentional design will shorten the time required to bring new materials to market and also enable the rational tailoring of transparency, conductivity, and mechanical flexibility for application specific needs. As the demand for smart, interactive, and energy-efficient devices grows, the further advancement of T/C chemistries, fabrication strategies, and AI-driven design will remain central to the future of optoelectronics.
